# Characterization of TCF‐1 and its relationship between CD8+ TIL densities and immune checkpoints and their joint influences on prognoses of lung adenocarcinoma patients

**DOI:** 10.1111/1759-7714.15058

**Published:** 2023-08-03

**Authors:** Kanqiu Jiang, Shasha Liu, Yongbing Chen, Zhonghen Xu, Zhonghua Xu, Bo Qian, Qifeng Ding, Hongxia Cui, Yiqun Sui, Defu Cao, Weihua Xu, Mingjing Shen

**Affiliations:** ^1^ Department of Thoracic and Cardiac Surgery The Second Affiliated Hospital of Soochow University Suzhou China; ^2^ Department of Rehabilitation Medicine The First Affiliated Hospital of Soochow University Suzhou China; ^3^ Gerontology Department Huadong Sanatorium Wuxi China; ^4^ Department of Pathology The Second Affiliated Hospital of Soochow University Suzhou China; ^5^ Department of Rehabilitation Medicine The Second Affiliated Hospital of Soochow University Suzhou China

**Keywords:** cluster of differentiation 8 (CD8), immune checkpoint inhibitors (ICs), lung adenocarcinoma (LUAD), prognosis, T cell factor‐1 (TCF‐1)

## Abstract

**Background:**

T cell factor‐1 (TCF‐1) + stem‐like tumor‐infiltrating lymphocytes (stem‐like TILs) are important memory cells in the tumor microenvironment. However, their relationship with clinicopathological features, CD8+ TIL densities, immune checkpoint inhibitors (ICs), and prognostic values remain unknown for lung adenocarcinomas (LUADs). In this study, we aimed to characterize TCF‐1+ TILs and their prognostic significance in patients with surgically resected LUADs.

**Methods:**

Expression of TCF‐1, CD8, and ICs including programmed death‐1 (PD‐1), lymphocyte activating‐3 (LAG‐3), and T cell immunoglobulin and mucin‐domain containing‐3 (TIM‐3) in TILs were estimated using immunohistochemistry of resected LUADs. The association between TCF‐1 expressions and clinicopathological characteristics of patient prognoses were analyzed.

**Results:**

Positive TCF‐1 expression significantly correlated with advanced pathological stage, tumor grade, CD8+ TILs density, TIM‐3 expression, LAG‐3 expression, and PD‐1 expression. TCF‐1 positivity was significantly associated with a better recurrence‐free survival (RFS), and overall survival (OS). Subgroup analysis revealed that the TCF‐1+/CD8+ group had the best RFS and OS, while the TCF‐1‐/CD8‐ group had the worst RFS and OS. Similarly, patients with TCF‐1 + PD‐1‐ had the best prognoses and patients with TCF‐1‐PD‐1+ had the worst prognoses.

**Conclusion:**

TCF‐1 had relatively high positive expression and special clinicopathological features in patients with LUAD. TCF‐1+ TILs were related to CD8 density, TIM‐3 expression, LAG‐3 expression, and PD‐1 expression, and were associated with better prognoses in LUAD patients. A combination of TCF‐1 and CD8 densities or PD‐1 expression further stratified patients into different groups with distinct prognoses.

## Graphical abstract

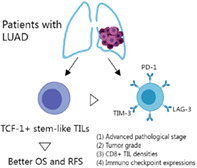

## INTRODUCTION

Lung cancer is the most common malignant cancer, which leads to a large number of deaths worldwide.^1^ Approximately 60% are lung adenocarcinomas, and most are diagnosed at a later stage.^2^, ^3^ Based on surgical resection findings, postoperative adjuvant therapy is usually required, which includes chemotherapy, radiotherapy, targeted therapy, and immunotherapy.^4^, ^5^, ^6^, ^7^, ^8^ Recently, immunotherapy has been improved due to the rapid clinical development of effective immune checkpoint inhibitors (ICIs) such as T lymphocyte‐associated antigen‐4 (CTLA‐4), programmed cell death 1 (PD‐1), and its ligand (PD‐L1).^9^, ^10^ In addition, other prospective immune checkpoints (ICs) such as lymphocyte activation gene‐3 (LAG‐3), T cell immunoglobulin and mucin‐domain containing 3 (TIM‐3), T cell immunoglobulin and ITIM domain (TIGIT), and V‐domain Ig suppressor of T cell activation (VISTA) are in preclinical trials.

Favorable outcomes of ICI treatments to a great extent depend on high infiltration of fully functional, cytotoxic effector TILs. Recently, a subset of TCF‐1+ stem‐like TILs were found to play vital roles in cancer immunotherapy. These progenitor cells sustain self‐renewal and proliferation during cancer development, which in turn helps maintain an antitumor response.^11^, ^12^ Transcription factor T cell factor 1 (TCF‐1), encoded by TCF‐7, is a critical transcription factor of TIL development. TCF‐1 silencing causes T progenitor cells to lose their self‐renewing ability, resulting in irreversible differentiation of effector TILs, as confirmed in mouse models.^13^

In previous studies, TCF‐1+ stem‐like TILs were associated with an ICI response in murine and human tumors.^14^, ^15^ The high infiltration of TCF‐1+ TILs has been shown to be associated with prolonged progression‐free survival (PFS) and overall survival (OS) in melanoma patients receiving checkpoint blockade.^16^ Ma et al. also reported the prognostic value of TCF‐1+ stem‐like TILs predicting better survivals in primary small cell carcinomas of the esophagus.^17^ However, the relationship between TCF‐1+ stem‐like TILs and clinicopathological characteristics and their prognostic value in patients with surgically resected lung adenocarcinoma is unknown. Moreover, TCF‐1+ TILs can undergo massive expansion in response to anti‐PD‐1 treatment,^14^ so the survival of combined TCF‐1+ stem‐like TILs and PD‐1 expression requires further research. In the present study, we therefore quantitatively analyzed clinicopathological features of TCF‐1 expression, evaluated its prognostic value, and assessed its associations with PD‐1, LAG‐3, and TIM‐3 expressions, as well as the density of CD8+ TILs.

## METHODS

### Patients and clinical pathology

A total of 350 patients with LUADs who were retrospectively treated with tumor resection in the Thoracic Department at The Second Affiliated Hospital of Soochow University from April 2015 to December 2018 were enrolled into the study. Some of the patients in our previous study were included in the cohort.^9^ The inclusion criteria of patients were as follows: (1) patients pathologically confirmed with primary LUAD according to the eighth edition of the TNM classification,^12^, ^14^ (2) patients who had not undergone preoperative neoadjuvant radiochemotherapy or targeted therapy and (3) patients with postoperative adjuvant chemotherapy based on cisplatin. The exclusion terms included: (1) patients lost to medical follow‐up, and (2) patients with other malignancies or concurrent multiple primary tumors. According to the criteria, 60 patients who accepted preoperative or postoperative chemoradiotherapy, targeted therapy, or immune therapy and 62 patients who were lost to follow‐up were excluded, with the remaining 228 patients enrolled. Immunohistochemistry (IHC) staining was used to detect the expressions of TCF‐1, PD‐1, LAG‐3, and TIM‐3 in specimens of these patients. Two experienced pathologists (YQS and HXC) who were blinded to the clinical outcomes, independently analyzed the IHC results. Discussions were made if there was controversy or discordance in the pathological diagnoses, followed by a consensus. LUADs were classified according to the IASLC/ATS/ERS classification^16^ and stages were determined according to the eighth edition of the TNM classification for LUAD.^18^, ^19^ The clinicopathological characteristics of these patients are summarized in Table 1, after an average follow‐up duration of 57 months. This study was approved by the Institutional Review Board of The Second Affiliated Hospital of Soochow University. Informed consent of patients was not required due to the retrospective nature of the study.

**TABLE 1:** Correlation between TCF1 expression and clinicopathologic parameters.

Variables	No. of patients	TCF‐1 expression		
		Positive (%)	Negative (%)	*p*‐value
Overall	228	48 (21.1)	180 (78.9)	
Age (year)				0.433
≤65	136	31 (22.8)	105 (77.2)	
>65	92	17(18.5)	75 (81.5)	
Sex				0.986
Male	138	29 (21.0)	109 (79.0)	
Female	90	19 (21.1)	71 (78.9)	
Smoking				0.973
Nonsmoker	124	26 (21.0)	98 (79.0)	
Current or former smoker	104	22 (21.2)	82 (78.8)	
Tumor location				0.269
Upper and middle lobe	146	34 (23.3)	112 (76.7)	
Lower lobe	82	14 (17.1)	68 (82.9)	
Pathological stage				0.045
I–II	153	38 (24.8)	115 (75.2)	
III	75	10 (13.3)	65 (86.7)	
CEA				0.126
≤10 ng/mL	188	36 (19.1)	152 (80.9)	
>10 ng/mL	40	12 (30.0)	28 (70.0)	
VPI				0.114
Absent	134	33 (24.6)	101 (75.4)	
Present	94	15 (16.0)	79 (84.0)	
Tumor grade				<0.001
Grade 1	32	14 (43.8)	18 (56.2)	
Grade 2	99	24 (24.2)	75 (75.8)	
Grade 3	97	10 (10.3)	87 (89.7)	
CD8 expression				0.036
Negative	161	28 (17.4)	133 (82.6)	
Positive	67	20(29.9)	47 (70.1)	
TIM‐3 expression				0.023
Negative	165	41 (24.8)	124 (75.2)	
Positive	63	7 (11.1)	56 (88.9)	
LAG‐3 expression				0.001
Negative	164	44 (26.8)	120 (73.2)	
Positive	64	4 (6.3)	60 (93.7)	
PD‐1 expression				0.002
Negative	163	43 (26.4)	120 (73.6)	
Positive	65	5 (7.7)	60 (92.3)	

Abbreviations: CEA, carcinoembryonic antigen; LAG‐3, lymphocyte activation gene‐3; No., number; PD‐1, programmed cell death‐1; TCF‐1, T cell factor 1; VPI, visceral pleural invasion; TIM‐3, T cell immunoglobulin and mucin‐domain containing‐3.

### Immunohistochemistry

Sections of tumor tissues were first deparaffinized and rehydrated. Endogenous peroxidase was then quenched using 10% H_2_O_2_ for 10 min at room temperature. Subsequently, 10% goat serum was used to block nonspecific proteins for 1 h. The sections were then rinsed and incubated with anti‐TCF‐1 (2203; diluted 1:150; Cell Signaling Technology), anti‐PD‐1 (ab55587; diluted 1:50; Abcam); anti‐TIM‐3 (ab241332; diluted 1:500; Abcam), or anti‐LAG‐3 (ab209236; diluted 1:500; Abcam) overnight at 4°C. Color development was conducted using the DAB horseradish peroxidase color development kit (Beyotime). Hematoxylin was used to counterstain sections before the final mount. When there was discordance, the final decision was made after discussion, using a multihead microscope.

### Determination of TCF‐1, PD‐1, LAG‐3, and TIM‐3 IHC cutoffs

For the determination of TCF‐1, a semi‐quantitative evaluation of TCF‐1 was performed using a previously described method.^20^ Staining intensity for TCF‐1 was scored as 0 (negative), 1 (weak), 2 (moderate), and 3 (strong). The degree of staining was scored as 0 (no staining), 1 (1%–25%), 2 (26%–50%), 3 (51%–75%), and 4 (76%–100%), which depended on the percentage of stained cells. The staining positivity was determined by the following formula: overall score = percentage score × intensity score. The total score ranged from 0 to 12, with negative staining (0–1) and positive expression (2–12) of TCF‐1. The cutoff score for PD‐1 was ≥8% staining according to a previous study.^21^ The cuoff value >20% was chosen for LAG‐3 to predict both recurrence‐free survival (RFS) and OS.^22^ We established the cutoff value for TIM‐3 in TILs as ≥11%, because the value accurately predicted the OS and RFS in LUADs.^23^

### Statistical analysis

Associations between clinicopathological characteristics were analyzed using the chi‐square or Fisher's exact test for categorical variables. In addition, a logistic regression model was used to confirm independent risk factors for the presence of TCF‐1. RFS was defined as the time from surgical resection to the first time of recurrence. OS was defined as the time from surgical resection until death from any cause or from the last follow‐up. RFS and OS were evaluated using the Kaplan–Meier method, and nonparametric group comparisons were performed using the log‐rank test. A Cox proportional‐hazards regression model was used to identify independent risk factors for RFS and OS. The variables were first examined using univariate analyses, and those with *p*‐values <0.05 were incorporated into a multivariate model. All *p*‐values were based on two‐tailed statistical analyses, and *p* < 0.05 was considered statistically significant. Statistical analyses were conducted using SPSS statistical software for Windows, version 25.0 (SPPS). The survival curves were drawn using Origin 2021 software (OriginLab).

## RESULTS

### Baseline information

The clinical characteristics of 228 patients are shown in Table 1. The mean age was 63 years (range: 30–79 years). A total of 138 (60.5%) were male and 90 (39.5%) were female. A total of 124 (54.4%) were smokers, and 104 (45.6%) were nonsmokers. The tumor locations of 146 (64.0%) patients were in the upper and middle lobes, and 82 (36.0%) tumors were located in the lower lobes. The range of tumor stages was broad; 153 (67.1%) were stages I and II and 75 (32.9%) were stage III. A total of 188 (82.5%) patients had carcinoembryonic antigen (CEA) levels lower than 10 ng/mL and 40 (17.5%) patients had CEA levels higher than 10 ng/mL. A total of 134 (58.7%) patients did not have visceral pleural invasion (VPI) and 94 (41.2%) had VPI. Patients were characterized until the last follow‐up or their death (median: 57 months).

### Characterization of TCF‐1 and its associations with clinicopathological factors of LUADs

Table 1 shows that TCF‐1 expression was more frequently identified in patients with advanced pathological stage (*p* = 0.045), by tumor grade (*p* < 0.001), and CD8+ TIL densities (*p* = 0.036). We did not detect any statistically significant difference in the associations between TCF‐1 expressions and age, sex, smoking history, tumor location, CEA levels in serum, and VPI.

### Relationships between TCF‐1 and ICs of LUADs

The relationships between TCF‐1 and ICs are shown in Table 1. High expression of TCF‐1 was significantly correlated with higher expression of TIM‐3 expression in TILs (*p* = 0.023), LAG‐3 expression in TILs (*p* = 0.001), and PD‐1 expression in TILs (*p* = 0.002).

### Expressions of TCF‐1, CD8, and IC

**FIGURE 1:**
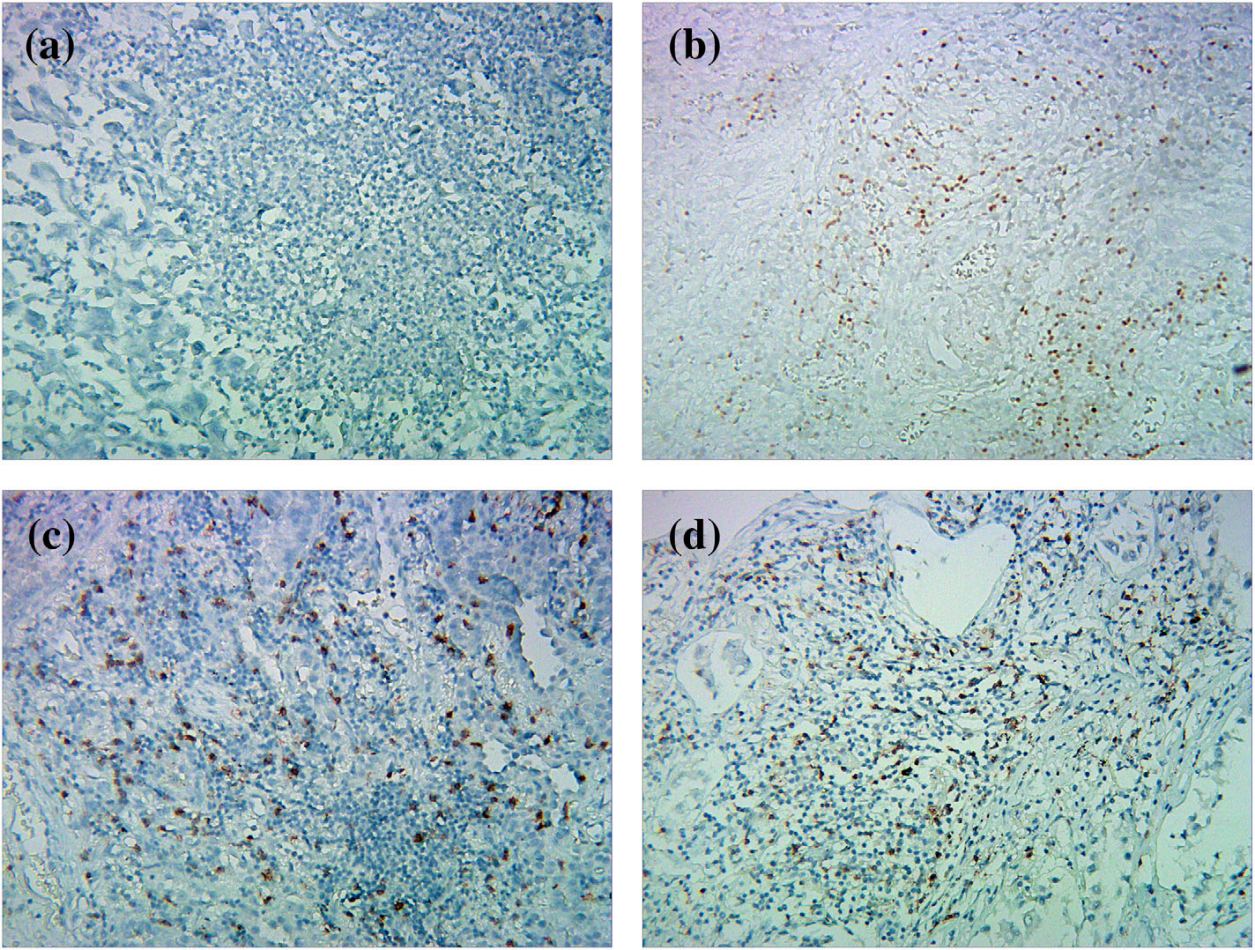
Negative and positive IHC staining for TCF‐1, CD8, and PD‐1 in TILs in the tumor microenvironment. (a) Negative expressions of TCF‐1, CD8, or PD‐1 in TILs. (b) Positive expression of TCF‐1 in TILs. (c) Positive expression of CD8 in TILs. (d) Positive expression of PD‐1 in TILs (magnification: 200×). IHC, immunochemistry; TCF‐1, T cell factor 1; CD8, cluster of differentiation 8; LAG‐3, lymphocyte activating 3; PD‐1, programmed death‐1; TIM‐3, T cell immunoglobulin and mucin‐domain containing‐3; TILs, tumor‐infiltrating lymphocytes; tumor microenvironment (TME).

IHC was performed to detect the expressions of TCF‐1, CD8, TIM‐3, LAG‐3, and PD‐1 in TILs. TCF‐1 positive expressions were detected in 48 (21.1%) patients, and CD8 positive expressions were detected in 67 (29.4%) patients. TIM‐3, LAG‐3, and PD‐1 in TILs were expressed in 63 (27.6%), 64 (28.1%), and 65(28.5%) patients, respectively (Table 1). Representative negative and positive stained fields of histopathological slides for TCF‐1, CD8, and PD‐1 are shown in Figure 1. IHC staining of TCF‐1, CD8, PD‐1, LAG‐3, and TIM‐3 were independently analyzed by two investigators. The agreement percentages were 92.1%, 90.8%, 87.8%, 89.2% and 91.0%, respectively.

### Multivariate logistic analysis to predict TCF‐1 expression

Multivariate logistic regression analyses showed that LAG‐3 positivity (odds ratio [OR]: 0.276; 95% CI: 0.091–0.841; *p* = 0.024), PD‐1 positivity (OR: 0.351; 95% CI: 0.125–0.986; *p* = 0.047), tumor grade 2 (OR: 0.294; 95% CI: 0.092–0.945; *p* = 0.040), and tumor grade 3 (OR: 0.136; 95% CI: 0.032–0.574; *p* = 0.007), were independent predictive factors for TCF‐1 expression (Table 2).

**TABLE 2:** Multivariate logistic regression model for TCF‐1 expression in patients with lung adenocarcinoma.

Variables	Multivariate analysis	
	OR(95%CI)	*p*‐value
CD8 (positive vs. negative)	0.933 (0.337–2.834)	0.966
TIM‐3 (positive vs. negative)	0.397 (0.154–1.024)	0.056
LAG‐3 (positive vs. negative)	0.276 (0.091–0.841)	0.024
PD‐1 (positive vs. negative)	0.351 (0.125–0.986)	0.047
Pathological stage (III vs. I–II)	0.418 (0.171–1.020)	0.055
Tumor grade		0.025
Grade 1	1	
Grade 2	0.294 (0.092–0.945)	0.040
Grade 3	0.136 (0.032–0.574)	0.007

Abbreviations: CI, confidence interval; LAG‐3, lymphocyte activation gene‐3; OR, odds ratio; PD‐1, programmed cell death‐1; TCF1, T cell factor 1; TIM‐3, T‐cell immunoglobulin and mucin‐domain containing‐3; vs., versus.

### Prognostic value of TCF‐1 expression and its combined effect with CD8 or PD‐1

**FIGURE 2:**
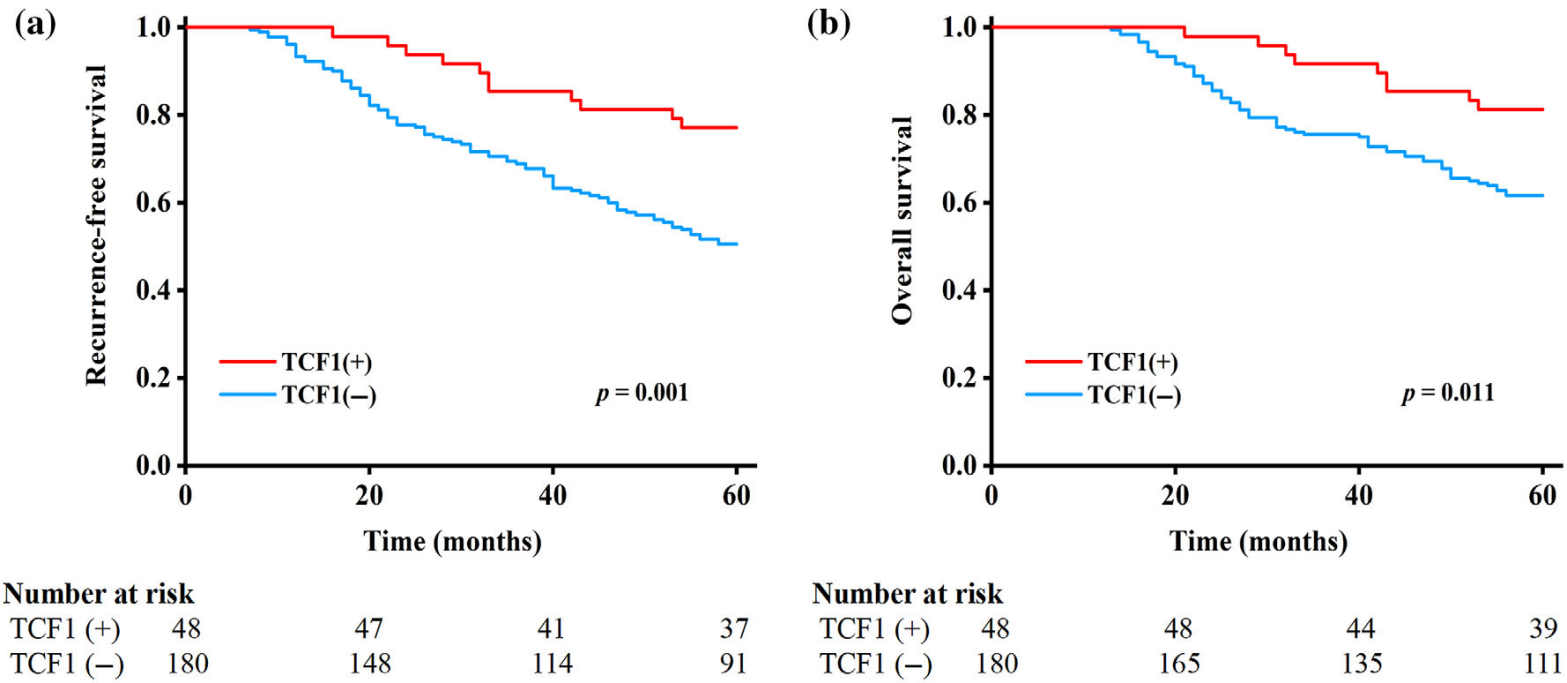
T cell factor 1 (TCF‐1) positivity, recurrence‐free survival (RFS), and overall survival (OS) in patients with lung adenocarcinomas. (a) RFS by TCF‐1, (b) OS by TCF‐1.

The log‐rank tests revealed that patients with TCF‐1 positivity had a significantly better RFS (5 year: 77.1% vs. 50.6%; *p* = 0.001) and OS (5 year: 81.3% vs. 61.7%; *p* = 0.011) compared with those with TCF‐1 negativity (Figure 2a,b).

**FIGURE 3:**
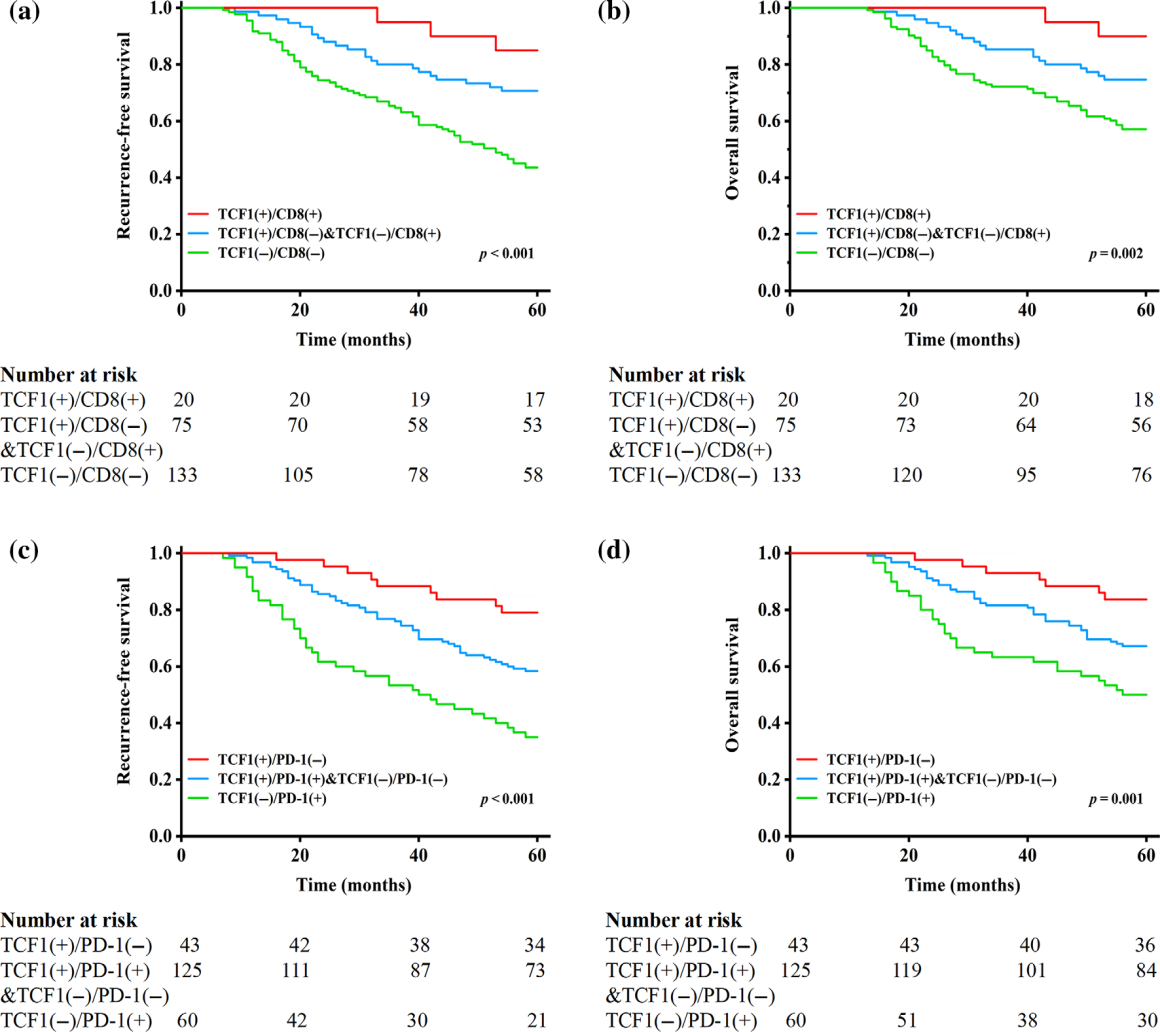
T cell factor 1 (TCF‐1), cluster of differentiation 8 (CD8), recurrence‐free survival (RFS), and overall survival (OS) in patients with lung adenocarcinoma (LUAD). (a) RFS by TCF‐1 and CD8. (b) OS by TCF‐1 and CD8. (c) RFS by TCF‐1 and programmed death‐1 (PD‐1). (d) OS by TCF‐1 and PD‐1.

We also determined the combined value of TCF‐1 with the prognoses of LUAD patients. Log‐rank tests revealed that patients with TCF‐1 + CD8+ had the best prognoses, patients with TCF‐1 + CD8‐ or TCF‐1‐CD8+ had moderate prognoses, and patients with TCF‐1‐CD8‐ had the worst prognoses (RFS: *p* < 0.001; Figure 3a; OS: *p* = 0.002, Figure 3b). Similarly, patients with TCF‐1 + PD‐1‐ had the best prognoses, patients with TCF‐1‐PD‐1‐ or TCF‐1 + PD‐1+ had moderate prognoses, and patients with TCF‐1‐PD‐1+ had the worst prognoses (RFS: *p* < 0.001; Figure 3c; OS: *p* = 0.001; Figure 3d).

### Cox regression analysis of RFS and OS

The variables of age, sex, smoking, tumor location, pathological stage, CEA level, VPI, pathological stage, tumor grade, TCF‐1 expression, CD8 expression, and IC expression were included in the univariate analyses, and survival associated variables in the Cox regression model were analyzed. Table 3 shows that TCF‐1 negativity (HR: 0.408; 95% CI: 0.188–0.886; *p* = 0.023), CD8 negativity (HR: 0.331; 95% CI: 0.111–0.986; *p* = 0.047), TIM‐3 expression positivity (HR: 1.680; 95% CI: 1.079–2.615; *p* = 0.022), LAG‐3 expression positivity (HR: 1.637; 95% CI: 1.038–2.581; *p* = 0.034), PD‐1 expression positivity (HR: 1.977; 95% CI: 1.264–3.094; *p* = 0.003), pathological stage (HR: 3.049; 95% CI: 1.670–5.568; *p* < 0.002), and tumor grade 3 (HR: 6.770; 95% CI: 1.261–36.344; *p* = 0.026) were independent prognostic factors due to a worsening RFS.

**TABLE 3:** Cox proportional‐hazards regression model for recurrence‐free survival and overall survival in all patients.

Variables	Recurrence‐free survival			Overall survival		
	Univariate analysis	Multivariate analysis		Univariate analysis	Multivariate analysis	
	*p*‐value	HR (95% CI)	*p*‐value	*p*‐value	HR (95% CI)	*p*‐value
Age (>65 vs. ≤65 years)	0.896			0.877		
Sex (male vs. female)	0.217			0.108		
Smoking (yes vs. no)	0.163			0.075		
Tumor location (upper and middle lobe vs. lower lobe)	0.565			0.685		
CEA (>10 vs. ≤10 ng/mL)	0.022	1.604 (0.816–3.153)	0.171	0.216		
VPI (present vs. absent)	<0.001	1.277 (0.665–2.451)	0.462	0.007	1.145 (0.627–2.091)	0.660
TCF‐1 (positive vs. negative)	0.001	0.408 (0.188–0.886)	0.023	0.011	0.410 (0.173–0.971)	0.043
CD8 (positive vs. negative)	<0.001	0.331 (0.111–0.986)	0.047	0.007	0.307 (0.096–0.982)	0.047
TIM‐3 (positive vs. negative)	0.014	1.680 (1.079–2.615)	0.022	0.014	1.765 (1.074–2.901)	0.025
LAG‐3 (positive vs. negative)	0.037	1.637 (1.038–2.581)	0.034	0.037	1.743 (1.037–2.931)	0.036
PD‐1 (positive vs. negative)	<0.001	1.977 (1.264–3.094)	0.003	0.001	1.941 (1.170–3.220)	0.010
Pathological stage (III vs. I–II)	0.013	3.049 (1.670–5.568)	<0.001	0.030	2.923 (1.669–5.118)	<0.001
Tumor grade	<0.001		0.004	<0.001		0.003
Grade 1		1			1	
Grade 2		2.295 (0.494–10.660)	0.289		1.617 (0.333–7.845)	0.551
Grade 3		6.770 (1.261–36.344)	0.026		5.809 (1.007–33.521)	0.049

*Note*: Smoking: current smoker or former smoker (yes) and never smoker (no). Variables with *p*‐value <0.05 in univariate models were analyzed in multivariate analysis model.

Abbreviations: CEA, carcinoembryonic antigen; CI, confidence interval; HR, hazard ratio; LAG‐3, lymphocyte activation gene‐3; PD‐1, programmed cell death‐1; TCF1, T cell factor 1; VPI, visceral pleural invasion; TIM‐3, T cell immunoglobulin and mucin‐domain containing‐3; vs., versus.

Moreover, TCF‐1 negativity (HR: 0.410; 95% CI: 0.173–0.971; *p* = 0.043), CD8 negativity (HR: 0.307; 95% CI: 0.096–0.982; *p* = 0.047), TIM‐3 expression positivity (HR: 1.765; 95% CI: 1.074–2.901; *p* = 0.025), LAG‐3 expression positivity (HR: 1.743; 95% CI, 1.037–2.931; *p* = 0.036), PD‐1 expression positivity (HR: 1.941; 95% CI: 1.170–3.220; *p* = 0.010), pathological stage (HR: 2.923; 95% CI: 1.669–5.118; *p* < 0.001), and tumor grade 3 (HR: 5.809; 95% CI: 1.007–33.521; *p* = 0.049) were independent prognostic factors for a worse OS.

## DISCUSSION

The past decade has witnessed the rapid development of immunotherapy for the treatment of cancer.^24^ Among these immunotherapies, use of coinhibitory immune ICIs, including PD‐1, PD‐L1, and CTLA‐4 monoclonal antibodies (mAbs), have become the most promising clinical treatments.^25^ The next generation of ICIs such as for LAG‐3, TIM‐3, TIGIT, VISTA, B7 homolog 3 protein, and B and T cell lymphocyte attenuators are now in preclinical trials. However, the responses of anti‐PD‐1/PD‐L1mAb or anti‐CTLA‐4 mAb is still far from satisfactory.^26^ Studies of novel ICIs have been ongoing. Meanwhile, quantitative detection of new and meaningful immunity‐associated proteins may help predict which patients will benefit from immunotherapy.

During progression of CD8+ T cell differentiation, a small subset of CD8+ T cells retain the potential for lymphoid recirculation and the ability of self‐renewal, resulting in the production of more differentiated effector TILs. These cells are defined by, and depend on, expression of the transcription factor, TCF‐1. This key transcription factor is essential for the generation of stem‐like TILs during cancer immunity.^27^, ^28^ Ablation of intratumoral TCF‐1+ TILs showed that TCF‐1‐ TILs lacked expansion capacity and restricted responses to immunotherapy. As a result, residual TILs lost their robust capability to maintain tumor control. In the past year, there have been reports documenting the presence of TCF‐1+ TILs in human cancers,^17^ and also reported results suggesting that the frequency of these cells was associated with their clinical outcomes. Miller and Sade‐Feldman reported that melanoma patients with high stem‐like TIL infiltration had a longer PFS and OS.^16^, ^29^ Accordingly, Ma et al. reported that primary small cell LUAD of esophagus patients with high infiltration of TCF‐1+ TILs had a longer OS and low infiltration (*p* = 0.009; HR: 0.506).^17^ In contrast, coexpression of LEF‐1 and TCF‐1 proteins in patients with nasopharyngeal carcinomas were positively correlated with lymph node metastasis (*p* = 0.001 and *p* = 0.020, respectively), advanced clinical stage (*p* < 0.003 and *p* = 0.027, respectively), and poor survival status (*p* < 0.001 and *p* = 0.004, respectively). In some other malignant tumors, TCF‐1 was overexpressed in osteosarcoma tissues, when compared with matched adjacent normal tissues.^30^, ^31^ Similar results have also been reported in renal cell carcinomas.^32^ The opposite role of TCF‐1 may depend on its location, which determines whether it is expressed in TILs or tumor cells. In the present study, TCF‐1+ TILs were more frequently found in LUAD patients with a higher tumor grade (*p* < 0.001), advanced pathological stage (*p* = 0.045), and greater CD8 expression (*p* = 0.036), but not age, sex, smoking history, tumor location, CEA level, or VPI. Further survival analyses revealed that LUAD patients with high TCF‐1 TIL infiltration had a higher RFS (*p* = 0.001) and OS (*p* = 0.011). Based on these results, we propose that infiltration of TCF‐1+ stem‐like TILs is a positive prognostic biomarker in LUAD patients.

Previous studies reported a proliferative burst of TILs after anti‐PD‐1 mAb treatment. Studies showed that these “newborn” TILs came exclusively from TCF‐1+ stem‐like TILs, so TCF‐1+ stem‐like TILs are critical for the effectiveness of ICI therapies. Man et al.^33^ reported T cell receptor‐induced transcription factors, IRF4, BATF, and NFATc1, promoted expression of inhibitory receptors, including PD‐1, and mediated decreased cellular metabolism. These transcription factors repressed the expression of TCF‐1. In contrast, inhibition of IRF4 expression restored the functional and metabolic properties of TILs and promoted memory‐like T cell development. TCF‐1+ stem‐like TILs, also called progenitor TILs or central memory TILs, differ from terminally exhausted TILs, which highly express PD‐1, TIM‐3, and LAG‐3. Consistently, our study confirmed that TCF‐1+ TILs in LUAD patients were negatively correlated with PD‐1 expression (*p* = 0.002), TIM‐3 expression (*p* = 0.023), and LAG‐3 expression (*p* = 0.001). In addition, multivariate logistic regression analyses showed that TIM‐3 expression (OR: 0.397; 95% CI: 0.154–1.024; *p* = 0.056), LAG‐3 expression (OR: 0.276; 95% CI: 0.091–0.841; *p* = 0.024), and PD‐1 expression (OR: 0.351; 95% CI: 0.125–0.986; *p* = 0.047), were independent risk factors for increased infiltration of TCF‐1+ TILs. These results implied the potential of TCF‐1 detection in predicting immunotherapy efficacy. This is similar to our previous study revealing the relationship between neutrophils in lung cancer microenvironment and ICIs expressions.^9^ The difference is that TCF‐1 had the negative correlation to ICIs expressions while neutrophils had an opposite tendency.

Because TCF‐1 acts as an important regulator in TIL stemness, studies have investigated the detailed gene axis and possible regulatory mechanisms. Chatterjee and Xu^34^, ^35^ reported that TCF‐1 was expressed in multiple isoforms in TILs, in which the long isoforms interacted with β‐catenin through an N‐terminal domain, while TCF‐1 short isoforms supported developing thymocytes to traverse through maturation steps to regulate most TCF‐1 target genes. Chemical inhibition of β‐catenin/TCF‐1 interactions improves long‐term self‐renewal and enhances functional pluripotency with increased Nanog expression. Man et al.^33^ showed that in CD8+ T cells, IRF4, BATF, and NFAT were recruited to adjacent binding sites, and binding of all three factors was significantly enriched among the core group of proteins related to exhaustion, including PD‐1, LAG‐3, HAVCR2, TIGIT, and CTLA‐4. Furthermore, Wu et al.^36^ reported that TCF‐1 acted upstream of the Bcl6‐Blimp1 axis in TCF‐1^high^ CD8 T cells, and that these TCF‐1^high^ CD8 T cells also expressed lower levels of canonical TH1 markers, including Blimp1 and Il2ra.^37^ IL‐2 signaling can repress TIL differentiation via the signal transducer and activator of transcription 5 (STAT5) pathways.^38^ In the present study, we found that positive TCF‐1 expression significantly correlated with expressions of PD‐1, LAG‐3, and TIM‐3, as well as high CD8 + TILs density. It is therefore possible that an internal connection between high expressions of coinhibitory ICIs and IRF4, BATF, and NFAT gene pathways in LUADs exists. Furthermore, the role of variable isoforms of TCF‐1 in promoting coinhibitory ICI expression should be investigated.

In conclusion, this is the first study to characterize TCF‐1 expression in TILs, and its prognostic significance in patients with surgically resected LUADs. However, there were some limitations in our study. First, performance and selection bias were inevitable because of the retrospective nature of the study. Second, we only included patients from a single institution. A prospective study and a larger cohort of patients with LUAD are therefore needed. Also, the patients lost to follow‐up appear high. Third, we did not investigate PD‐L1, an established predictive marker for immunotherapy. Additional multicenter studies with larger patient cohorts may address these limitations.

In conclusion, TCF‐1+ TILs had relatively high positive infiltrations and specific clinicopathological features in patients with LUADs. TCF‐1+ TILs significantly correlated with pathological stage, tumor grade, CD8+ TILs density, and PD‐1, LAG‐3, and TIM‐3 expression levels in TILs. TCF‐1+ TILs were significantly associated with a better RFS and OS. Furthermore, the combination of TCF‐1+ TILs and PD‐1 or CD8 expression in TILs further stratified patients into distinct groups with different prognoses.

## AUTHOR CONTRIBUTIONS

Conceptualization, methodology and writing original draft: K. J, B. Q, Data curation and writing original draft preparation: Y. C, Visualization and investigation: S. L, Resources and visualization: Z. X, Software and validation: H. C, Writing‐reviewing and editing: Z. X, Validation formal analysis: Y. S, Resources: D. C, Investigation: W. X, Software formal analysis: Q. D, Conceptualization and supervision project administration: M. S.

## FUNDING INFORMATION

This work was supported by the Technological Innovation Project of CNNC Medical Industry Co. Ltd (ZHYLYB2021007), the Hospital Internal Research Foundation for Talent Cultivation (XKTJ‐RC202010), the Suzhou Science and Technology Foundation (SKJY2021077 & SKY2022166), the Hospital Internal Research Foundation for PhD (SDFEYBS2009), the Preresearch Fund Project of the Second Affiliated Hospital of Soochow University (SDFEYQN1915), the Medical Technology Program of Suzhou National Hi‐Tech District (2019Q015), the Jiangsu Key Research and Development Plan (Social Development) Project (BE2020653), the Discipline Construction Project of the Second Affiliated Hospital of Soochow University (XKTJ‐XK202004), and the Gusu Health Talent Program of Suzhou (GSWS2021020 & GSWS2022147).

## CONFLICT OF INTEREST STATEMENT

The authors declare no conflicts of interest.
